# Lateral Extraarticular Tenodesis in Revision Anterior Cruciate Ligament Reconstruction: An Analysis of Clinical Outcomes and Failure Rates

**DOI:** 10.3390/jcm13237201

**Published:** 2024-11-27

**Authors:** Lorenz Fritsch, Luca Bausch, Armin Runer, Philipp W. Winkler, Romed P. Vieider, Sebastian Siebenlist, Julian Mehl, Lukas Willinger

**Affiliations:** 1 Department of Sports Orthopaedics, Technical University Munich, Ismaningerstr. 22, 81675 Munich, Germanyl.willinger@tum.de (L.W.); 2 Department for Orthopaedics and Traumatology, Kepler University Hospital GmbH, Johannes Kepler University Linz, 4020 Linz, Austria

**Keywords:** LET, revision ACL surgery, knee laxity

## Abstract

**Background/Objectives**: Lateral extraarticular tenodesis (LET) has been advocated in revision anterior cruciate ligament reconstruction (ACLR) to improve knee stability and furthermore, decrease failure rates. The aim of this study was to compare clinical outcomes, knee laxity, and failure rates after revision ACLR with LET (ACLR + LET) versus without LET. It was hypothesized that ACLR + LET improves clinical outcomes and reduces the failure rate. **Methods**: A retrospective analysis of prospectively collected data was conducted to examine patients who underwent revision ACLR with and without LET between 2017 and 2021 with a minimum follow-up of 24 months. Patients with coronal malalignment (>5°), posterior tibial slope >12°, and concomitant injuries to collateral ligaments were excluded. Patient reported outcome measures (PROMs) included the International Knee Documentation Committee (IKDC) subjective knee score, the Lysholm score, and the Tegner activity scale (TAS). Anterior knee laxity was measured with a Rolimeter and side-to-side difference (SSD) was determined. Revision ACLR failure was defined as ACL revision surgery and SSD > 5 mm. Group comparisons were performed using chi-square-, independent-samples students *t*-test or Mann–Whitney–U tests. **Results**: Of 56 eligible patients, 45 (80%, follow up, 23 isolated rACLR vs. 22 rACLR + LET) were included with a mean follow-up of 45.6 ± 15.8 months. Postoperative PROMs were not statistically different between rACLR and rACLR + LET groups (IKDC: 73.0 ± 18.9 vs. 68.7 ± 16.6, n.s.; Lysholm: 84.8 ± 12.3 vs. 77.7 ± 16.2, n.s.). Both groups showed similar TAS (rACLR vs. rACLR +LET (5; range 4–6 vs. 4; range 3–5; n.s.). Anterior knee laxity SSD was 2.4 ± 1.3 mm in the rACLR group and 1.8 ± 1.8 mm in the rACLR + LET group (n.s.). The failure rate was 13% in the rACLR group compared to 4.5% in the rACLR + LET group (n.s.). **Conclusions**: Isolated revision ACLR showed comparable postoperative patient-reported outcome measures and anterior knee laxity compared to ACLR + LET at mid-term follow up. The addition of a LET demonstrated a lower, though non-significant, failure rate after revision surgery. However, future studies with a prospective, randomized design and an increased number of patients are needed to clearly identify the exact indication for the use of additional LET.

## 1. Introduction

Failure after anterior cruciate ligament reconstruction (ACLR) is devastating and there is a high scientific interest in identifying risk factors and surgical strategies to reduce re-ruptures. The failure rate of primary ACLR remains high, at 3–20% [1,2,3], and in a selective patient collective of up to 30% [3], with an odds for ACL re-rupture of 3.6 for highly active patients in comparison to less active patients [4]. Revision ACLR is demanding and shows unfavorable clinical outcomes and even higher failure rates of 5–20% than in the primary ACLR [1,5,6,7].

An increased tibial slope >12° [8,9,10,11,12,13], concomitant meniscal, and collateral ligament injuries, an increased anterolateral rotatory knee laxity [14,15], coronal malalignment [16], and an increased medial laxity [17] have been described to increase the re-rupture risk.

In contrary, there exists strong evidence that an additional anterolateral stabilization procedure, such as a lateral extraarticular tenodesis (LET) or anterolateral ligament reconstruction, decreases the re-rupture risk after ACLR [18,19,20,21,22,23]. The LET provides a load-sharing function with the ACL and protects the ACL graft from overload from disrupting forces [24,25]. Also, in revision ACL surgery, the literature demonstrated a favorable effect of additional LET on the graft survival [15,26], especially in a highly active patient cohort [27]. Furthermore, the recent literature demonstrated that patients being treated with additional LET in revision ACLR are more likely to return to their prior sportive level than patients with isolated revision ACLR [23,28]. The protecting and stabilizing effect of the LET has been widely accepted. However, it introduces additional surgical risks for patients and alters the anatomy of the anterolateral soft tissue. Current data are limited regarding the subjective mid-term clinical outcome, knee laxity, and rates to return to sports (RTS) comparing revision ACLR with or without additional LET procedures [6,28,29]. Evidence remains scarce whether LET has an influence on the subjective patients’ outcome and the ability to return to sports at mid-term follow up.

The aim of this study was to compare patient reported outcomes, knee laxity, and failure rates in patients who underwent revision ACLR with or without LET. It was hypothesized that an additional LET leads to similar clinical outcome scores with a decreased failure rate.

## 2. Materials and Methods

This retrospective analysis of prospectively collected data was approved by the insti-tutional ethical review board and conducted according to the Declaration of Helsinki, and approved by the Institutional Review Board of Technical University Munich (protocol code: 2023-296-S-SR and date of approval: 30 June 2023). All patients gave their informed consent to participate in this study. Patients older than 18 years who either underwent revision ACL reconstruction or revision ACLR with an additional LET between February 2017 and December 2021 with a minimum follow-up of 24 months were eligible for inclusion. Exclusion criteria obtained varus or valgus malalignment >5°, posterior tibial slope >12°, as well as reconstruction of the medial or lateral collateral ligament or any type of chondral therapy. Patients with meniscal injuries were included, as they were present in the majority of eligible patients. Patients with enlarged, malpositioned or semianatomic bone tunnels had primary bone tunnel filling and received revision ACLR during a second surgery after 3 months.

Surgical technique: The revision ACLR was performed in a single-bundle technique using either autologous quadriceps or a 4-fold hamstring tendon, depending on what graft was used in the primary reconstruction. The femoral tunnel drilling was performed through the anteromedial portal aiming at the anteromedial bundle position. The tibial tunnel was placed in the anatomical footprint medial to the anterior horn of the lateral meniscus. Femoral fixation of the ACL graft was performed with an extracortical suspensory fixation system (Tight Rope, Arthrex, Naples, FL, USA). Tibial graft fixation was carried out with a bio-interference screw one size larger than the drill tunnel (Arthrex, Naples, FL, USA). The indication for LET was upon the surgeon’s preference.

The LET was either performed in a modified Lemaire technique [30] or as a modified Ellison technique [31,32]. Until the end of 2020, the modified Lemaire technique was primarily performed. Afterwards, the modified Ellison technique was preferred as tunnel conflicts with the ACL reconstruction could be more easily avoided. The patient is placed in supine position with the operated site bent in 90° of flexion. The surgical approach for both procedures was equivalent: anatomical landmarks in terms of Gerdy’s tubercle and the femoral condyle. The skin incision is made 1 cm proximal to the lateral femoral epicondyle leading to Gerdy’s tubercle for an optimal approach to the ITB. For the Lemaire technique, a 10 mm stripe of the ITB was cut over a length of approximately 8 cm. It was passed beneath the lateral collateral ligament (LCL) and fixed femoral with a bio-interference screw posteriorly and proximally to the lateral epicondyle. In contrast, the stripe of the ITB was similarly prepared and shuttled from proximal to distal below the LCL with it being fixed on Gerdy’s tubercle for the Ellison technique [31,32].

The postoperative protocol was equal in both groups. For all patients, a hinge brace was applied for six weeks. For all patients who received an isolated rACLR, 20 kg of weight was applied for two weeks after surgery. Patients with concomitant meniscal repair were limited to six weeks of a 20 kg weight. Early physiotherapy focused on restoration of full extension and thigh muscle activation.

Six weeks postoperatively, therapy focused on muscular strengthening; cycling was allowed after eight weeks and running was allowed after twelve weeks. High-risk sports, with pivoting and cutting maneuvers, such as football or basketball, were not allowed nine months after surgery.

Clinical evaluation: At a minimum follow-up of 24 months, patients were invited and clinically examined to assess the range of motion (ROM) and knee laxity by the means of Lachman- and pivot-shift tests and were rated according to the IKDC knee examination form. The anterior tibial translation (ATT) was measured in 30° of knee flexion in neutral and internal tibial rotation, trying to evaluate the effect of LET on prohibiting tibial internal translation, using a Rolimeter (Aircast Europa, Neubeuern, Germany) and compared to the healthy contralateral knee by calculating the side-to-side difference (SSD).

PROMs included the International Knee Documentation Committee Subjective Knee Form (IKDC), the Lysholm score, Tegner activity scale (TAS), and visual analogue scale (VAS) for pain. Revision surgeries were also documented. RTS was defined as the return to any level of sports participation based on the preoperative TAS. Consequently, the change in sports activity was calculated by comparing the pre -to postoperative TAS; return to sports was defined as return to activity and the return to same level was defined as reaching the same level of preoperative TAS.

Additionally, all patients completed a questionnaire including satisfaction on a 10-point Likert scale (10 = very satisfied, 0 = very unsatisfied) as well as patient reported outcome measures (PROMs).

Statistical analysis: The statistical analysis was performed using the SPSS-software version 26.6 (IBM, New York, NY, USA). Based on die minimal clinically important difference (MCID) of ACL reconstruction (differences of mean = 10 ± 11), a sample size calculation resulted in a total number of 42 patients with a 1:1 allocation to detect a difference in mean of the IKDC score between patients with isolated ACLR and combined ACLR + LET at a critical *p*-value of 0.05 with an actual power of 81.9% [33]. Descriptive statistics were used to summarize categorical and continuous variables, with categorical variables reported as counts and percentages, and continuous variables reported as mean ± standard deviation or median and the interquartile range (IQR). A Kolmogorov–Smirnov test was used to evaluate normal distribution. An unpaired *t*-test was utilized to compare differences in means. A side-to-side difference (SSD) of more than 5 mm was considered as surgical failure as previously described [26]. Odds Ratio (OR; confidence interval 95%) was calculated to describe the probability of potential re-rupture or re-instability; Fisher’s exact test was used to examine the failure rate. Binary logistic regression was used to calculate the risk for re-rupture including the age, BMI, and LET. Statistical significance was set to *p* < 0.05.

## 3. Results

**Figure 1:**
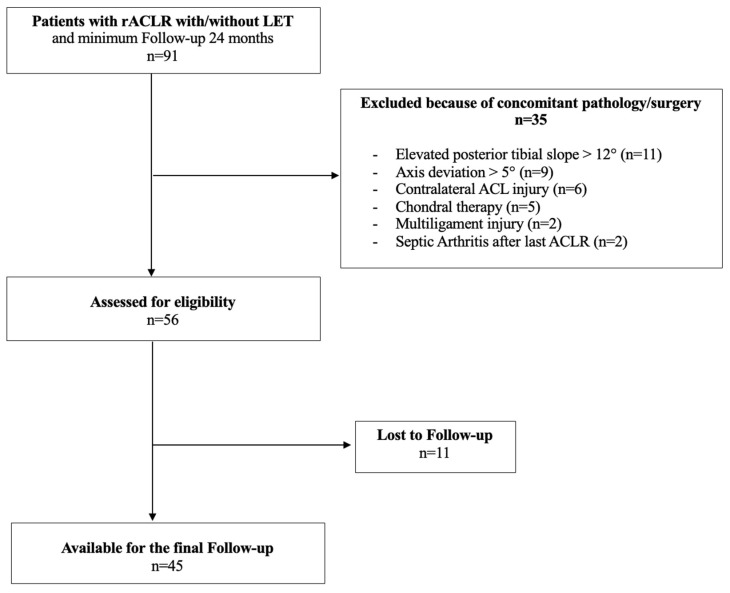
Flowchart of eligible patients with the number of excluded patients because of additional risk factors/concomitant injuries.

In total, 45 of 56 (80.4%) patients were included after a mean follow-up of 48.6 ± 16.3 (range 24–66 months) months. Twenty-three patients were allocated in the isolated revision ACLR group and 22 patients in the group combined revision ACLR with LET group. The groups did not differ in demographic parameters and preoperative sport activity level (Table 1 and Table 2). A total of 28 patients had a single staged revision surgery; 17 patients underwent a two-staged revision surgery with primary filling of the bone tunnels. Excluded patients or patients lost to follow-up are presented in Figure 1.

Further demographic and surgical characteristics of the patient population are provided in Table 1.

Clinical outcome: Clinical outcome scores are provided in Table 2. Obtained PROMs were not significantly different between the study groups.

Return to sports rate: The postoperative TAS was significantly lower compared to preoperative values in the rACLR group (*p* < 0.001) and the rACLR + LET group (*p* < 0.001). The RTS rate was 86.7% in the rACLR group (30.4% returned to the same or higher level) and 95.5% in the rACLR + LET group (9.1% returned to the same or higher level).

Complications and failures: Four patients underwent revision surgery due to recurrent laxity or ACL re-rupture: three failures (two atraumatic, one traumatic) occurred in patients of the rACLR group treated with a quadriceps tendon graft (13.1%). There was one failure reported in the rACL + LET group in a patient treated with a hamstring tendon graft + LET (4.5%, OR: 3.1, confidence interval 95%; *p* = 0.09). In a binary regression analysis, no significant difference was found for age, BMI, or the use of a LET on the failure of ACL revision surgery (*p* = n.s.). No other complications were reported.

Clinical evaluation: Patients who were not re-revised were included in the clinical examination. There was no restriction in ROM in either group, with all patients achieving full extension and at least 130° of knee flexion. In the rACLR group, eight patients showed a Lachman grade I. In the rACLR group combined with LET, 10 patients exhibited a Lachman grade I. Regarding the anterior drawer test, two patients in the rACLR group had a grade I, while six patients in the rACLR group + LET showed a grade I. For the pivot shift test, one patient in the rACLR group had a grade I, compared to two patients in the rACLR group + LET.

Knee laxity: Regarding the Rolimeter testing, there was no significant difference in ATT between the study groups (Table 3). ATT in internal rotation was smaller in both groups compared to neutral rotation (*p* < 0.05). None of the patients exhibited an SSD of more than 5 mm compared to the healthy side.

## 4. Discussion

The main findings of this study were that similar clinical outcome scores and adequate anterior knee stability resulted after either isolated revision ACLR or combined revision ACLR with LET. There was a tendency that combined revision ACLR and LET showed lower outcome scores and a lower revision rate; however, this was not statistically significant. High satisfaction was observed in both cohorts and patients could return to an adequate level of activity which was significantly lower compared to the preoperative sports level.

In primary ACL-reconstruction, additional LET significantly reduces the rate of graft failure. In a study by Getgood et al., 436 patients were examined after isolated ACL reconstruction and ACL reconstruction with additional LET [18]. Patients were prospectively collected in several centers, randomly allocated to the groups, and followed over a period of two years. At the final follow-up, both cohorts returned to a similar level of sports; however, additional LET significantly reduced the risk for graft failure.

Adding a LET has also been proven to be beneficial to reduce the failure rate in revision ACLR [15,19,26,34,35]. Alm et al. showed a significant reduction in revision ACLR failure from 21.4% to 5.1% by adding a LET in modified Lemaire technique after 2 years follow up [26]. Similarly, Helito et al. and Trojani et al. demonstrated a reduced failure rate after the addition of an anterolateral procedure compared to isolated revision ACLR [34].

In a systematic review, Saithna et al. [19] reported the failure rates in revision ACLR + LET as in the range of 0–13% and in isolated revision ACLR in the range of 4–21%, which is comparable to the present study. It is important to mention that there was no difference in failure rate in patients with low-grade anterolateral rotatory knee laxity [5]. Furthermore, Vivacqua et al. [36] compared patients after revision ACLR with and without LET and did not detect a reduction in graft failure when using autologous quadriceps or patella tendon grafts. 

With regard to postoperative anterior knee laxity, there is evidence that an additional LET decreases postoperative rotational and translational knee laxity [5,22,23,26,34]. Four studies found a significant reduction in ATT by adding a LET compared to isolated revision ACLR [22,26,34]. The mean SSD for anterior knee laxity was between 1.3 and 3.9 mm in patients with a combined procedure and between 1.8 and 5.9 mm in patients with an isolated revision ACLR [19]. In contrast, Pineda et al. [37] performed stress x-rays of primary ACL reconstructions with and without LET. However, no significant difference concerning the SSD was noted between the two cohorts, meaning that the combined ACLR and LET was not fully able to restore native anterior tibial translation under the physiological axial load. In the present study, no difference was observed in the anterior tibial translation in neutral rotation and internal rotation when using the Rolimeter for measurement. The remaining SSD of ATT was similar to former studies measuring 1.8–2.4 mm. There is also a trend towards a higher postoperative rotatory laxity in patients with isolated ACLR [1,11,16,35]. However, only one study was statistically significant [11]. In this study, no high-grade pivot shift (grade II or III) was observed. 

Concerning subjective clinical outcome, the majority of previously published studies reported the IDKC and Lysholm score to be in favor of the combined revision ACLR and LET procedure [1,4,11,16,17,35]. Alm et al. [1] demonstrated a superior postoperative Lysholm score and IKDC score when an additional LET was performed in comparison with isolated revision ACLR. Also, the postoperative Lysholm score was higher in revision ACLR + LET compared to an isolated ACLR [1,11]. In contrast, no differences in the outcome scores were observed with even lower absolute values in the additional LET group in the present study cohort. 

Overall, the RTS rate after revision ACLR is high, in the range of 48–88% [25] with no study being able to show a significant difference between the groups [4,15,16]. This is in agreement with our findings, which showed no difference in the RTS rate. Grassi et al. [9] mentioned 74% to be able to return to sports after additional LET. However, only 41% of their cohort was able to return to the same level prior to the ACL injury. Several studies reported the postoperative TAS and showed a mean postoperative TAS between 4 and 7 in both groups. Only one study reported a significant difference favoring the ACLR + LET group [16]. The patients in the present study adapted their preoperative activity level after mid-term follow up from an average of 7 to 4. This may be explained due to the fact that the cohort mostly consists of recreational athletes. Winkler et al. [33] showed that multiple revision led to a lower TAS of 4 compared to the first revision ACLR who achieved a TAS of 6.

## 5. Limitations

First, both groups consisted of a small number of patients; therefore, the possibility of a statistical error type II must be considered, especially for the statistical significance of clinical outcome scores and the failure rate. Second, the study inherits the bias of a retrospective data collection of the PROMs. Further, the indication for LET was not based on objective criteria, but rather on the surgeon’s preference. The study patients were not matched, however, there was no difference in the demographic data or preoperative activity level which makes the groups comparable. Third, patients with meniscal injuries were included, but the distribution was equal in both groups. Lastly, the presence of osteoarthritis, which is highly prevalent in patients with ACL insufficiency, was not included in the final analysis, though it could contribute to an inferior outcome.

## 6. Conclusions

Additional LET in revision ACLR leads to a tendency of a reduced risk of failure with similar clinical outcomes in comparison to isolated revision ACLR. However, both groups revealed favorable clinical outcomes and a low failure rate. The rate of RTS is high with patients returning to a lower level of activity compared to preoperative values. However, future studies with a prospective, randomized design and an increased number of patients are needed to clearly identify the exact indication for the use of additional LET.

## Figures and Tables

**Table 1:** Demographic and surgical data of the included patients showing no differences between the study groups. Values reported as mean ± standard deviation; *p* < 0.05.

		rACLR	rACLR + LET	*p*-Value
Sex (N)				
	Male	13	14	0.82
	Female	10	8	0.65
Age (years)		30.5 ± 8.9	27.2 ± 9.4	0.26
Body mass index (kg/m^2^)		26.8 ± 6.1	24.5 ± 3.4	0.12
Follow-up (months)		51 ± 16.4	46.1 ± 15.9	0.19
Graft type (N (%))				
	Quadriceps tendon	17 (74%)	14 (64%)	0.7
	4-fold hamstring tendon	6 (26%)	8 (36%)	0.67
Concomitant surgeries (N)				
	Partial resection of the medial meniscus	13	6	0.1
	Medial meniscus repair	12	7	0.09
	Partial resection of the lateral meniscus	1	1	1.0
	Lateral meniscus repair	6	4	0.67

**Table 2:** Clinical outcomes were not statistically significantly different between the rACLR group and the rACLR + LET group. Values reported as mean ± standard deviation (SD) and median and interquartile range (IQR).

	rACLR	rACLR + LET	*p*-Value
IKDC	73.0 ± 18.9	68.7 ± 16.6	0.43
Lysholm score	84.8 ± 12.3	77.7 ± 16.2	0.14
Preoperative TAS	7 (6–7)	7 (5–8)	0.43
Postoperative TAS	5 (4–6)	4 (3–5)	0.20
VAS	2.0 ± 1.9	2.1 ± 2.1	0.88
Satisfaction rate	9 (7–10)	8 (7–9)	0.65

**Table 3:** The knee laxity tested with a Rolimeter device (Aircast Europa, Neubeuern, Germany) showed a similar anterior tibial translation given as side-to-side difference to the contralateral healthy knee in the rACLR group and the rACLR + LET group. Values reported as mean ± standard deviation (SD); *p* < 0.05.

	rACLR	rACLR + LET	*p*-Value
Neutral tibial rotation (mm)	2.4 ± 1.3	1.8 ± 1.8	0.22
Internal tibial rotation (mm)	1.6 ± 1.2	1.0 ± 1.4	0.10

## Data Availability

The raw data supporting the conclusions of this article will be made available by the authors on request.
